# Psychological Well-Being, Marital Satisfaction, and Parental Burnout in Iranian Parents: The Effect of Home Quarantine During COVID-19 Outbreaks

**DOI:** 10.3389/fpsyg.2020.553880

**Published:** 2020-12-03

**Authors:** Seyyedeh Fatemeh Mousavi

**Affiliations:** Department of Psychology, Women Research Center, Alzahra University, Tehran, Iran

**Keywords:** well-being, parental burnout, marital satisfaction, home quarantine, COVID-19

## Abstract

Coronavirus disease 2019 (COVID-19), as an infectious disease, is now prevalent in many countries around the world, which has recently led many governments to home quarantine and impose penalties for violating quarantine. Concerns and stress caused by lockdown and social isolation led to personal and interactive reactions in some families, which are also culturally important to address. This study was administrated to study the psychological well-being and the effect of home quarantine on marital satisfaction (MS) and parental burnout (PB) from parenting responsibilities during the prevalence of COVID-19 in Iranian parents. A total of 213 parents (140 mothers and 73 fathers) voluntarily participated in the online survey in the period of February to mid-April 2020 and completed the 5-item index of the well-being of the World Health Organization (WHO-5), the Kansas Marital Satisfaction Scale (KMSS), and the Parental Burnout Assessment (PBA). The results showed that the effect of home quarantine on MS and PB was not significant in parents (*p* > 0.01). The interactive effect of home quarantine and gender was not significant on MS and PB (*p* > 0.01). In addition, the mothers significantly reported higher PB than the fathers, but the fathers had higher scores in MS and psychological well-being (*p* < 0.01). The effect of some demographic factors on the studied variables was also significant. Supportive resources in family-based culture may play an essential role to reduce the negative effects of stressful situations on family interactions.

## Introduction

Coronavirus disease 2019 (COVID-19), as an infectious disease, is now recognized as a highly prevalent disease that affects a large number of people, especially the elderly and people with a background of health problems (World Health Organization [WHO], 2020). Since no definitive treatment has been discovered so far to treat this prevalent disease, the only way to cope with the disease and cut off the virus transmission chain is to do personal hygiene and keep physical distance from others in daily interactions. This has led governments to quarantine people at home and impose penalties for violating these laws. This type of home quarantine is called lockdown, and with the onset of the COVID-19 outbreak, millions of people around the world are living in conditions of social isolation and constraint on social interactions (Taub, 2020).

In Iran, after closing all public places and forcing people to stay at home and not moving in public places, people’s interactions were limited. This time almost coincided with the beginning of the Iranian New Year. Since Iranian culture is a culture based on family and kinship interactions, it imposed severe restrictions on an individuals’ access to relatives and friends’ networks; worry, death anxiety, and feelings of hopelessness were among the concerns raised in people’s contacts with the counseling section of the Welfare Organization (Welfare Organization of Iran [WOI], 2020). Such a condition, as an external, major, non-normal, out of control and unpredictable stressor to cope, has been unprecedented in the history of the world and has led to different forms of self-isolation and can cause different behavioral and emotional reactions (Gallagher, 2020) in different individuals with different cultural backgrounds. Such psychological reactions due to the decline of social relations are more evident in traditional cultures; researchers have shown that decreased emotional contact and feelings of connection with family and friends are associated with symptoms of distress and depression in African-Americans (Taylor et al., 2016, 2020). Recent studies have shown that social distancing by creating a sense of loneliness, anxiety, suicide ideations (Armbruster and Klotzbücher, 2020), low perception of social support, and an inability to connect with others is associated with low well-being and low sense of social connectedness (Emerson et al., 2020; Garcia et al., 2020). In addition to the personal effects of the lockdown, home quarantine and social distancing challenged the value and the cost of relationships with others. In such situations, people accept staying at home and distancing from friends and family as the cost of maintaining their physical health, while with self-exclusion of the social network, they were imposed lots of psychological harm. However, the side costs of staying at home for a long time, such as multiplying the caring responsibilities of spouse and children (Long, 2020), are also a matter of consideration.

Due to the novelty of studies on the personal and interactional effects of home quarantine caused by the coronavirus outbreak, the current study was conducted to investigate the effect of home quarantine on psychological well-being and marital and parental outcomes in families living in Iran.

### Stressful Life Events and Psychological Well-Being

Mental health has been defined as the absence of mental disorder (which enables a person to fulfill the personal growth and experiences of happiness and satisfaction) (World Health Organization [WHO], 2001). Thus, psychological well-being is an integrated process apart from mental disorder that involves a concept of more than happiness and includes a concept of optimistic development (Fen et al., 2013). Psychological well-being is the first element that is threatened in the face of stressful life events. Stressful life events with dimensions including challenging the adaptive mechanisms of the individual (Holmes and Rahe, 1967), being threatened (Cohen et al., 2016), imposing demands on the individual over accessible resources (Lazarus and Folkman, 1984), and disrupting the achievement of life goals (Kemeny, 2003) have many physical and psychological consequences for a person (Cohen et al., 2019). Cleland et al. (2016) showed that stressful life events, such as losing a good relationship, health problems, and economic problems, have negative impacts on mental health. Following some epidemic economic crises, negative social consequences, including declining economic well-being, physical and psychological problems, and the adoption of relevant coping strategies, are predictable (Aytaç et al., 2015). The prevalence of an unknown and epidemic disease, such as COVID-19, as a stressful event that affects a large number of people (Delle Fave, 2014) reduces the psychological well-being (Philip and Cherian, 2020; Sibley et al., 2020), threatens social relationships, social confidences, and maintaining connections (Delle Fave, 2014), weakens the socio-economic position of the individual, loses a sense of control over life events, and reduces the feeling of life satisfaction (Rubio et al., 2018), especially when it was considered as an unpredictable and chronic disease, and when it occurs for at-risk groups, such as the elderly or people with emotional disorders (Schneiderman et al., 2005; World Health Organization [WHO], 2009).

The prevalence of the unknown coronavirus disease as a stressful event, in addition to direct effects on health, due to forcing people to quarantine at home can have several psychological consequences on individuals. Limited studies in this area show that lockdown and being isolated have significantly increased feelings of loneliness, anxiety, stress, and suicide in people living in European and American countries (Brodeur et al., 2020). Other evidence suggests that children in quarantine situations experience feelings of anger, fear, loneliness, and boredom despite feeling happy and relaxed with family (Idoiaga Mondragon et al., 2020), Conversely, the study of Recchi et al. (2020) on the French population before and after the outbreak of coronavirus indicated an improvement in well-being and health in the unaffected majority; however, people with low income and who lost their job or those who worked long hours at home reported higher levels of stress (Prickett et al., 2020).

### Stressful Life Events, Lockdown, and Parental Burnout

The home quarantine and lockdown situation caused by the COVID-19 crisis have affected the well-being of families and challenged the ability of parents to carry out parenting responsibilities by imposing more education and care responsibilities (Mangiavacchi et al., 2020). The closure of schools and some businesses imposed many housework and childcare responsibilities on parents, especially on mothers (Farré et al., 2020). Alon et al. (2020) and Hupkau and Petrongolo (2020) showed in a survey of United States and United Kingdom families that housework, childcare duties, and the role of teaching for children have put more pressure on parents, especially on mothers. Stressful situations by influencing people’s coping strategies are associated with the high negative mood symptoms of depression, anxiety, and stress in parents (Mather et al., 2014; Flouri et al., 2018), symptoms of burnout (Mather et al., 2014), and parent–child dysfunctional interactions (Platt et al., 2016). Some studies have shown that lack of control, household workload, role confusion, and irrational environmental demands especially caused by stressful events, such as the COVID-19 pandemic (Griffith, 2020), have led to negative emotions, such as exhaustion, feelings of helplessness, frustration, and finally parental burnout (PB) in caregivers (Vinayak and Dhanoa, 2017; Mikolajczak et al., 2018a).

Parental burnout is a state of emotional exhaustion that is associated with a change in positive attitudes toward children (Luescher et al., 1999) and is defined in four dimensions as the feeling of exhaustion of parental role leading to low parental efficiency, feeling of fed up, contrast with the past parental role, and emotional distancing from children (Roskam et al., 2018). Studies have shown that PB is associated with parent–child demographic factors, such as the age of mother and child, the number of children at home, the number and gender of caregiver, socio-economic status of the family, the physical and mental conditions of parent–child (Blanchard et al., 2006; Norberg, 2007; Lindström et al., 2011; Vinayak and Dhanoa, 2017; Le Vigouroux and Scola, 2018; Mikolajczak et al., 2018b; Mousavi et al., 2020), personal factors, such as emotional intelligence and high parental self-efficacy beliefs, and interactional factors, such as positive parenting practices, co-parenting, and marital satisfaction (MS) (Mikolajczak et al., 2018b).

### Stressful Life Events, Lockdown, and MS

Marital satisfaction as the indicator of the marriage quality (Fincham and Beach, 1999) is defined as the mental experience of happiness in a marital relationship (Hendrick et al., 1998), which reflects the perceived benefits and values of marriage (Baumeister and Vohs, 2007), a subjective evaluation that varies under the influence of happy and unpleasant life events (Li and Wickrama, 2014). The challenging events in the context of the family are changes, and people no longer simply feel satisfied after the changes (Maltas, 1992); changes caused by stressful events led to stress and act as a threat to satisfaction and intimate relationships (Randall and Bodenmann, 2009). Stressors, especially external factors, first by reducing opportunities to strengthen relationships and distracting couples from spending time to improve intimacy in relationships and then by weakening their abilities to cope with stressors, create problems for couples (Neff and Karney, 2017). Some studies have shown that external stressors including natural disasters are associated with decreased MS (Chi et al., 2011). Studies have also shown that stressful events (e.g., declining level of welfare and low income) have a significant association with decreasing marital stability (Bahr, 1979), poorer marital interactions and low MS, especially in the first years of marital life (Bodenmann, 2000; Bodenmann and Cina, 2006), sexual problems in intimate relationships (Bodenmann et al., 2007), criticism of spouse, and reduction of dyadic support (Neff and Karney, 2009), low MS, and high psychological distress with mediating role of problem-solving (Cohan and Bradbury, 1997) or management skills (Li and Wickrama, 2014) and coping strategies (Fink and Shapiro, 2013).

The prevalence of the COVID-19 pandemic as an external stress has challenged the quality of couples’ relationships; home quarantine with negative psychological effects, such as anxiety, stress, depression, and frustration (Brodeur et al., 2020; Idoiaga Mondragon et al., 2020), and making changes in the unemployment rate and reducing access to financial opportunities (Coibion et al., 2020), forcing distress couples to spend many hours of the day together and increase the capacity for marital conflicts (Perez-Vincent et al., 2020), can have negative effects on couples’ relationships; these effects become more destructive due to different backgrounds, such as low economic quality and low levels of vulnerability (Pietromonaco and Overall, 2020). Kevin and Risla (2020) showed that couples with low income reported low MS during the lockdown or distressed couples experience worse individual and dyadic outcomes in the face of external stress (Beach et al., 1994). Conversely, some other evidence suggests that external stresses in a good marital relationship, despite weakening the relationship at the beginning of the encounter to stress, cause spouses to adapt to new conditions and strengthen the dyadic relationship in later stages (Bonanno, 2004). Therefore, to clearly understand the effect of stress on couples’ relationships, it is helpful to pay attention to the source of stress, the intensity of stress, and the duration of exposure to stress (Randall and Bodenmann, 2009) with regard to personal, relational, and cultural backgrounds of couples.

### Gender Differences in Well-Being, PB, and MS

Gender plays an essential role in the distribution of role and power in the family and social status (World Health Organization [WHO], 2019). High non-stop parental and household tasks, the monotony of endless daily affairs, and the low level of control in mothers can be an explanation for the poor mental health compared with fathers (Baruch et al., 1987). This is why the prevalence of emotional disorders is higher in women than in men (World Health Organization [WHO], 2019). In other studies, mothers reported more stress and anxiety, less well-being (Skreden et al., 2012; Meier et al., 2018; Nelson-Coffey et al., 2019), less happiness, more fatigue, and consequently, less leisure time and sleep (Musick et al., 2016), which is explained by the more limited role of fathers in parenting and social isolation (Skreden et al., 2012).

Many studies have shown that it is the mothers who, as the primary and permanent caregivers of the children, are more exposed to PB than the fathers (Le Vigouroux and Scola, 2018; Lebert-Charron et al., 2018; Mikolajczak et al., 2018b; Roskam et al., 2018; Roskam and Mikolajczak, 2020). Mothers, especially those who have young children or more children or who do not have the capacity of fathers to cooperate in parenting responsibilities, suffer from more PB (Hubert and Aujoulat, 2018; Mikolajczak et al., 2018b). Mothers also felt more burned out than fathers because of high parenting expectations and social pressures for being a perfect mother (Meeussen and Van Laar, 2018; Sorkkila and Aunola, 2020) and were characterized by feelings of guilt, anxiety, and severe depression (Sánchez-Rodríguez et al., 2019), although fathers reported more negative consequences for PB, such as suicidal ideation, running away, and child neglect (Roskam and Mikolajczak, 2020).

Although parenting in mothers is associated with more reports of burnout (Roskam et al., 2018; Roskam and Mikolajczak, 2020), having children is one of the hallmarks of MS in mothers (Twenge et al., 2003). Beam et al. (2018) attributed the gender difference in MS to the difference in the structure of their marital quality. Olson et al. (2008) found that women were more likely to complain than men from distancing to successful marital life standards and were more sensitive to marital problems. Job satisfaction, communication, mutual understanding, and sexual satisfaction were reported as important components of MS in men and the same factors except for sexual satisfaction in MS in women (Ayub and Iqbal, 2012). In the study by Ogolsky and Bowers (2013), maintenance behaviors were reported to lead to MS in women more than in men, and women’s MS was greater when their husbands accompanied them in spending time with their children and supported them in caregiving (Rhyne, 1981; Leung, 2020). Therefore, increasing MS in mothers is associated with increased participation of fathers in the home and high family cohesion (Korja et al., 2016; Liu and Wu, 2018) and parent–child interactions (Yoo, 2020).

### Current Study

The unexpected occurrence of infectious disease of COVID-19, the coercion of people into home quarantine, in addition to hearing the news of daily deaths, preventing people from holding mourning ceremony for lost loved ones, closing schools and businesses, and the resulting economic pressure on low-income groups, and so on, caused anxiety and seeing an uncertain future ahead, that the only way to cope with this phenomenon was to distancing from the community for cutting off the chain of disease outbreaks. The pressure of new circumstances on individual and relationship outcomes in Iranian families led the researcher to evaluate the effects of the coronavirus outbreak on well-being, marital outcomes, and parent–child interactions by investigating the families’ perceptions of the pre–post outbreak of coronavirus condition. Hence, to assess MS and PB before and after the COVID-19 outbreak (home quarantine condition), the researcher presented both scales into two sections with past and present tense verbs (perception of MS and PB in the pre-outbreak condition and how they are feeling now about their marital and parental outcomes) to test the research hypotheses as follows:

*Hypothesis 1*: The home quarantine due to the COVID-19 outbreak has increased burnout and reduced MS among Iranian parents.

*Hypothesis 2*: The PB and MS scores of mothers and fathers are significantly different in the pre–post outbreak of COVID-19.

*Hypothesis 3*: The mothers and fathers are significantly different in well-being, PB, and MS scores.

## Materials and Methods

### Participants

Due to the study of marital and parental outcomes, the statistical population of the current study includes all married fathers and mothers living in cities of Iran who have at least one child who needs care at home (without age limit), are literate, and have consented to participate in the study. From 215 parents who participated in this study with the exclusion of two mothers who had experienced death caused by COVID-19 in the family, a total of 213 parents remained, of whom 140 only mothers and 73 only fathers participated throughout Iran, and 91 parents were from Tehran (capital of Iran), and the rest of them were from different cities. The demographic characteristics of *mothers* were: age (37.43 ± 7.22), the length of the marriage (14.46 ± 8.14), the number of children (1–4 child), the level of education (up to graduation of high school = 38, bachelor = 49, MA/MS = 33, Ph.D. = 20), have paid activities (*N* = 67) or not having (*N* = 73), and welfare level of the neighborhood (non-prosperous = 6, average = 107, prosperous = 27).

The demographic characteristics of *fathers* were: age (41.95 ± 8.6), the length of the marriage (15.64 ± 10.7), the number of children (1–6 child), the level of education (up to graduation of high school = 6, bachelor = 24, MA/MS = 31, Ph.D. = 12), have paid activities (*N* = 72) or not having (*N* = 1), and welfare level of the neighborhood (non-prosperous = 4, average = 56, prosperous = 13).

### Procedure

The present study is cross-sectional and has a mixed factorial design. Therefore, this study has a within-subjects variable (home quarantine) with two facets (before and after the COVID-19 outbreak) and a between-subjects variable (gender) with two facets (fathers and mothers). The study was conducted for nearly 1 month and a half after the outbreak of COVID-19 that forced people to home quarantine in cities of Iran. Therefore, to evaluate the effect of home quarantine, the items of the scales were presented to the participants in both past and present tenses and in two sections including before and after the outbreak; thus, the research had two limitations: (1) the impossibility of conducting longitudinal research to evaluate the effect of home quarantine on the studied variables and (2) the impossibility of conducting a survey in paper–pencil to reach a high sample size; the online survey was used. The survey was administrated online (an Iranian online survey website^1^) from last February to mid-April 2020 for nearly a month and a half past the prevalence of COVID-19 in Iran and coincided almost with the beginning of the New Year (Nowruz Holiday) in Iran. The link of the survey was shared accompanied by a video clip introducing the research goal on social media including WhatsApp and Telegram, and so on. The project took almost a month to complete, and data analysis began after no one was added to the project for a week. All participants voluntarily participated in the online survey and were free to withdraw from participation. They were assured of the confidentiality of the information and the use of the results for academic research.

### Measures

*Demographic information* includes participants’ information about the gender, the age of parents, the length of the marriage, the number of children, the level of education, have or not having paid activities, the welfare level of the neighborhood, and the experience of death due to COVID-19 in the family.

*Open-ended questions* are used to evaluate how the families react to hearing about outbreaks and deaths of COVID-19 and how to cope with this stress as well as how they spent time at home with their children. These questions were used for explaining the quantitative results, such as what is used in explanatory sequential designs (Wisdom and Creswell, 2013), and the well-being and parental outcomes of parents were described in details in the face of the COVID-19 outbreak and home quarantine.

*Marital satisfaction* is measured by the Kansas Marital Satisfaction Scale (KMSS). This 3-item self-reported scale assesses couples’ satisfaction with the relationship, partner as a spouse, and marriage in a 7-point Likert scale from *extremely satisfied* (1) to *extremely dissatisfied* (7) (Nichols et al., 1983). Based on a meta-analysis study about MS tools, this scale had the highest reliability (Graham et al., 2011). Its convergent validity with the Relationship Assessment Scale (RAS) has been reported as 0.74 in Iranian couples (Dehshiri and Mousavi, 2015a). This scale is also used in two sections before and after the COVID-19 prevalence using the past (for example, “How satisfied were you with your relationship with your husband (or wife)?”) and present tense verbs (for example, “How satisfied are you with your relationship with your husband (or wife)?”). In this study, Cronbach’s alpha coefficient (before and after the outbreak) was obtained as 0.94 and 0.97, respectively.

*Parental burnout* is assessed by Parental Burnout Assessment (PBA). This self-report scale includes 23 items in a 7-point Likert scale from *never* (0) to *every day* (6) that was prepared by Roskam et al. (2018) to assess parental exhaustion from parenting responsibilities in four subscales: exhaustion of the parental role, contrast with the parental self in the past, feeling of being fed up, and emotional distancing from children. The scale was validated in Iranian by Mousavi et al. (2020), and Cronbach’s alpha was obtained at the range of 0.60–0.93 for subscales. Participants were asked to express their perceptions of experiencing burnout before and after the COVID-19 outbreak that forced home quarantine in two sections with past (for example, “I couldn’t stand my role as father/mother anymore.”) and present tense verbs (for example, “I can’t stand my role as father/mother anymore.”). The Cronbach’s alpha for the perceived PB before the COVID-19 outbreak was obtained at the range of 0.76–0.97, and after the outbreak it was at the range of 0.87–0.98. Since one of the points in the 7-point scale was “a few times a year” (6) and was not applicable for the post-outbreak condition, this point was removed from the scale, and it became a 6-point Likert scale (0–5). Like any disorder, PB naturally does not have normal distribution and positively skewed, so, to observe the assumption of normality of the dependent variable for doing any parametric test, the natural logarithm conversion was used to normalize the scores.

*Psychological well-being* is assessed by the 5-item index of the well-being of the World Health Organization (WHO-5). This self-report scale is based on the WHO-10 scale that was prepared by Blom et al. (2012) and assessed the well-being of a person over the past 2 weeks on a 6-point Likert scale from *at no time* (0) to *all of the time* (5). Therefore, this scale was used in one section to assess the well-being of mothers and fathers in this situation (for example, “Over the last 2 weeks: I have felt calm and relaxed.”). Higher scores indicate higher well-being, and lower scores show poorer well-being of a person. In a study to validate this scale in Iranian people, Dehshiri and Mousavi (2015b) found a Cronbach’s alpha coefficient of 0.89. In this study, its Cronbach’s alpha coefficient was obtained at 0.89.

### Data Analysis

After reporting the mean and standard deviation of well-being, PB, and MS, a mixed factorial design was used to assess PB and MS before and after outbreak conditions in both genders. To assessing its assumption, the normality test was applied. The skewness and kurtosis of well-being scores (−0.45 and −0.18) and the skewness (−0.155 and −1.8) and kurtosis (2.69 and 3.39) of MS scores showed that the distribution of scores is normal. Since the skewness (2.97 and 2.79) and kurtosis (7.54 and 7.41) of PB were not normal, so for the normalization of scores, the natural logarithm conversion was used, and the skewness (0.67 and 0.77) and kurtosis (−0.77 and −0.6) of PB were obtained. The Levene’s Test results in MS scores also showed that the assumption of homogeneity of variance [*F*(1,211) = 1.07, *p* > 0.3; *F*(1,211) = 2.91, *p* > 0.9] was satisfied. However, the Levene’s Test in examining the homogeneity of variance of PB scores showed that the variance of the two groups is not equal; based on variance ratio, the ratio of the biggest variance to smallest variance is less than 3, so, the variance of the two groups can be considered equal (Field et al., 2012).

To analyze the relationship between well-being, PB, and MS (in the present condition, i.e., after the outbreak) with other demographic variables, Pearson correlation analysis and one-way ANOVA were used in which the assessing of the assumptions of both tests showed, respectively, that the relationship between two variables was indicated as linear by the scatter plot and the normality of dependent variables distribution and the homogeneity of variances of dependent variables were satisfied.

In addition, the analysis type of open-ended questions was by content analysis that was done manually by analyzing, categorizing, and coding the themes in the responses of 213 participants. In coding the responses using inductive or open coding method, first, after getting a sense of all the answers to each question, a sample of data was read, and based on the themes, they were categorized, and their codes were assigned in Excel sheet; this process was continued until all the participants’ responses were coded, similar responses were placed in pre-assigned codes, and new codes were assigned to new responses. Finally, all the categories and codes were reviewed to ensure that similar codes can be merged, and some irrelevant responses were also removed. In total, out of three open-ended questions, 256 responses and 5 codes were assigned to the first question (how to react to the news of the coronavirus outbreak); 601 responses were analyzed, and in two coding steps, 11 and 3 codes were assigned, respectively, to the second question (how to reduce stress); and 528 responses were analyzed, and in two coding steps, 22 and 4 codes were assigned, respectively, to the third question (how to spend time with children).

## Results

*Analysis of the responses to short questions*: In analyzing the psychological reaction to hearing the news about the outbreak of COVID-19, 45.6% reported that they are trying to be calm and relax, 22.13% showed anxiety, 16.60% showed stress, 12.56% showed fear and apprehension, and 3.56% reported no reaction. For reduction of stress, participants also reported doing the following activities: prayer, talk to GOD, and strengthen religious beliefs with 34.45%, housework (cooking and baking bread and cookies) and doing art and handicrafts with 20.80%, and the expansion of virtual communication with family, friends, and relatives with 11.98%. For spending free time with the children, parents also reported the following activities: planning intellectual and group games and competitions at home with 73%, monitoring school assignments and playing the role of teacher for children with 8.52%, experience a variety of cooking and confectionery for them with 7.01%, and playing virtual and computer games with 5.87%.

The descriptive statistics including mean and SD in Table 1 show the scores of parents in well-being, MS, and PB according to gender and time periods, the level of education, and have or not having paid professional activities.

**TABLE 1:** Mean and SD of wellbeing, marital satisfaction and parental burnout according to gender, education, and job before and after home quarantine.

	Gender						Education								Job			
			
							Up to graduation of											
	Mothers (*N* = 140)		Fathers (*N* = 73)		Total (*N* = 213)		high school (*N* = 44)		Bachelor (*N* = 73)		MA/MS (*N* = 64)		Ph.D. (*N* = 32)		With paid (*N* = 139)		Not paid (*N* = 74)	
									
	Pre	Post	Pre	Post	Pre	Post	Pre	Post	Pre	Post	Pre	Post	Pre	Post	Pre	Post	Pre	Post
	*M (SD)*	*M (SD)*	*M (SD)*	*M (SD)*	*M (SD)*	*M (SD)*	*M (SD)*	*M (SD)*	*M (SD)*	*M (SD)*	*M (SD)*	*M (SD)*	*M (SD)*	*M (SD)*	*M (SD)*	*M (SD)*	*M (SD)*	*M (SD)*
Wellbeing	15.76 (5.41)		17.93 (5.34)		16.50 (5.47)		17.05 (5.9)		15.68 (5.81)		16.98 (5.3)		16.66 (4.31)		17.29 (5.43)		15.01 (5.29)	
Marital satisfaction	16.86 (4.008)	17 (4.38)	18.11 (3.7)	18.37 (3.56)	17.29 (3.93)	17.47 (4.16)	17.07 (4.49)	17 (5.03)	17.18 (4.2)	17.38 (4.42)	16.98 (3.73)	17.19 (3.82)	18.47 (2.7)	18.88 (3.37)	17.69 (3.58)	18.01 (3.51)	16.54 (4.48)	16.64 (5.02)
Parental burnout	1.54 (1.51)	1.52 (1.53)	0.94 (1.29)	1.03 (1.23)	1.33 (1.47)	1.35 (1.46)	1.3 (1.62)	1.27 (1.55)	1.61 (1.54)	1.58 (1.51)	1.11 (1.25)	1.07 (1.29)	1.24 (1.44)	1.49 (1.42)	1.16 (1.42)	1.21 (1.4)	1.67 (1.51)	1.61 (1.52)

### The Effect of Home Quarantine and Gender on MS, PB, and Well-Being

In testing Hypothesis 1, the analysis of variances in Table 2 showed that the effect of home quarantine was not significant on MS, *F*(1,211) = 1.32, *p* > 0.25, η^2^ = 0.006, and PB too, *F*(1,211) = 0.26, *p* > 0.61, η^2^ = 0.001.

**TABLE 2:** Analysis of variance of MS and PB before and after COVID-19 outbreak.

	**Source of variation**	***SS***	**df**	***MS***	***F***	***p***	**η*^2^***
Marital satisfaction	**Between subjects**						
	Gender	164.07	1	164.07	5.6	0.02	0.26
	Error	6184.32	211	29.31			
	**Within subjects**						
	Home quarantine	3.76	1	3.76	1.32	0.25	0.006
	Home quarantine * gender	0.37	1	0.37	0.13	0.72	0.001
	Error	620.24	211	2.85			
Parental burnout	**Between subjects**						
	gender	28.86	1	28.86	7.8	0.006	0.36
	Error	780.92	211	3.7			
	**Within subjects**						
	Home quarantine	0.17	1	0.17	0.26	0.61	0.001
	Home quarantine * gender	0.32	1	0.32	0.71	0.4	0.003
	Error	96.26	211	0.456			

**FIGURE 1:**
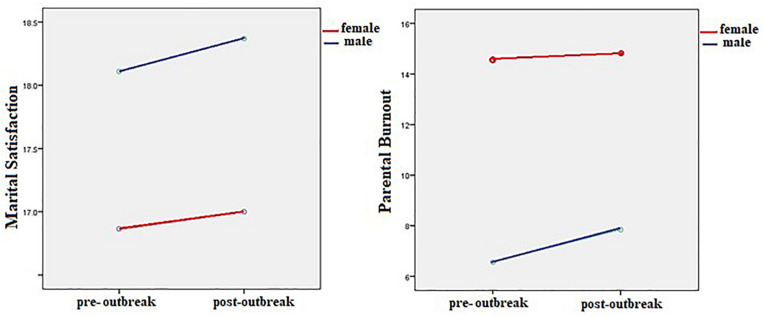
MS and PB of parents before and after the COVID-19 outbreak.

Table 2 also shows that the interactive effect of home quarantine and gender was not significant on MS, *F*(1,211) = 0.13, *p* > 0.72, η^2^ = 0.000, and PB, *F*(1,211) = 0.71, *p* > 0.49, η^2^ = 0.003. Figure 1 shows the effect of home quarantine across the genders.

In examining Hypothesis 3, after controlling the effect of pre-prevalence, the result in Table 2 also revealed that the gender effect for both of the two variables MS, *F*(1,211) = 5.6, *p* < 0.02, η^2^ = 0.26, and PB, *F*(1,211) = 7.8, *p* < 0.006, η^2^ = 0.36, was significant. The mean comparison in Table 1 showed that the fathers (18.37 ± 3.56) have reported more MS than the mothers (17 ± 4.38) and lower burnout (1.03 ± 1.23) than the mothers (1.52 ± 1.53). The ANOVAs showed that gender has a significant effect on well-being too, *F*(1,211) = 7.82, *p* < 0.01, η^2^ = 0.19. A comparison of the means of the two groups of mothers and fathers in Table 1 shows that the mothers (15.76 ± 5.41) reported poorer well-being than the fathers (17.93 ± 5.34).

### Additional Analysis With Demographic Factors

The results showed that there was no significant relationship between the *length of marriage* and well-being (*r* = −0.05, *p* > 0.4) and PB (*r* = −0.09, *p* > 0.19), but a significant negative relationship with MS (*r* = −0.15, *p* < 0.02). The results showed that there was no significant relationship between the number of children and well-being (*r* = 0.10, *p* > 0.1) and PB (*r* = −0.09, *p* > 0.1) and MS (*r* = −0.005, *p* > 0.9). In addition, no significant relationship was observed between parents’ *age* and well-being (*r* = 0.04, *p* > 0.6) and PB (i = −0.1, *p* > 0.7) and MS (*r* = −0.09, *p* > 0.1).

The results of ANOVAs showed that parents were not significantly different in terms of *education* in well-being *F*(3,209) = 0.86, *p* > 0.5, η^2^ = 0.012, MS *F*(3,209) = 1.53, *p* > 0.2, η^2^ = 0.021, and PB *F*(3,209) = 1.51, *p* > 0.2, η^2^ = 0.021; but in terms of *job*, parents having a paid profession had higher scores on well-being *F*(1,211) = 8.7, *p* < 0.004, η^2^ = 0.4 and MS *F*(1,211) = 6.89, *p* < 0.009, η^2^ = 0.3, but did not differ significantly in PB *F*(1,211) = 3.57, *p* > 0.06, η^2^ = 0.017 rather to ones not having a paid profession. In terms of *neighborhood* welfare, parents were significantly different in the scores of well-being and PB, the results showed that parents living in average neighborhood reported higher well-being than parents living in disadvantaged neighborhood (Δ*M* = 4.47, *p* < 0.04), parents living in prosperous neighborhoods reported the highest scores in well-being compared with the other two groups (Δ*M* = 6.8, *p* < 0.002), and parents living in prosperous neighborhoods reported lower scores on PB than parents living in average neighborhoods (Δ*M* = −0.69, *p* < 0.03).

## Discussion

Crises caused by epidemics lead to many psychological reactions in individuals. The coronavirus epidemic caused by acute respiratory syndrome, which has spread to many Asian, European, and American countries, has become a global public health concern. The disease has affected many people so far and has led to the death of a number of people in each country, depending on the health facilities and financial and supportive resources; due to the possibility of recurrence and lack of vaccines or definitive drugs for treatment (so far), the only mechanism is restricting the social interactions of people, which led to many closed centers, in addition to severe disruption in the economy and negative psychological outcomes in people around the globe.

The current study was administrated to examine the effect of the outbreak and home quarantine on psychological well-being, PB, and MS of Iranian mothers and fathers. The results showed that home quarantine did not have a significant effect on PB. Although studies in the field of PB are still in their infancy, some of these studies have shown that negative life events, increased environmental demand, problems due to poverty, unemployment, and low economic status, and stress, such as lack of physical and mental health of children and parents, can predict parental exhaustion (Blanchard et al., 2006; Norberg, 2007; Mikolajczak et al., 2018b). Accordingly, the finding of this study is not consistent with these studies. Iranian society is a family-based society in which many sources of support come from the family. Hence, many individual and interactive outcomes can be explained and interpreted in the context of the family. According to the parents’ answers to the open questions, it can be seen that the parents for leisure time during the quarantine period provided different activities, such as planning intellectual and group games and competitions at home and playing the role of coach for children as compensation for absence at school; such activities have involved children in various activities, so, staying at home instead of threatening seems to provide a good capacity to pay more attention to housework and children.

Despite the insignificance of the interaction between home quarantine and gender, we can still see an increase in the average scores of fathers in burnout compared with mothers after the outbreak of the disease and staying at home. The presence of fathers at home and their involvement in the performance of household chores and children, although an opportunity to reduce the pressure of permanent and pre-defined parenting and housekeeping duties for mothers and also compensates for the lost educational opportunities of the children, but due to the lack of experience of fathers being at home so much and the division of such responsibilities for them in home quarantine conditions, led to the exhaustion of the fathers; this can be a matter of concern, especially in traditional cultures, such as Iran, where traditional gender roles are defined and accepted for men. The findings by Mangiavacchi et al. (2020) also showed that the closure and lockdown, by restricting the taking of the children to nursing centers, has led to positive changes in the involvement of fathers in caring and educational tasks of children at home and increasing children’s well-being. Such involvement of fathers has also been seen, given the distribution of gender roles in European countries, such as Italy (Barigozzi et al., 2020).

The results also showed that the home quarantine does not have a significant effect on MS. This finding is inconsistent with the results of some studies based on the effect of external stress on MS reduction (Maltas, 1992; Neff and Karney, 2009, 2017; Randall and Bodenmann, 2009; Chi et al., 2011). New studies on the effects of coronavirus and lockdown outbreaks on family interactions have shown that the lockdown has weakened the marital interactions by increasing the rate of unemployment and reducing access to financial resources (Coibion et al., 2020), especially in troubled couples (Perez-Vincent et al., 2020). The findings by Kevin and Risla (2020) indicated the association between low income and reduced MS, especially in distressed couples. Other findings of this study showed that parents having a paid profession and are living in prosperous neighborhoods reported higher MS and well-being and lower PB. On the other hand, MS as a couple’s perception of the multidimensions of marital life and the perceived benefits of a long-term relationship was formed over months and years of communication and affected by different factors, such as life skills, understanding and intimacy, and the feeling of security in the relationship, and so on (Fincham and Beach, 1999; Baumeister and Vohs, 2007). Couples with well-functional interactions support each other and use dyadic coping strategies in coping with external sources of stress (Randall and Bodenmann, 2009), as well as problem-solving techniques and marital life management skills as dyadic coping behaviors have shown a moderating role in the effects of the stressful events on marital quality (Cohan and Bradbury, 1997; Li and Wickrama, 2014). Despite the limitation of the current study in collecting data from distressed couples, an increase in the MS scores of mothers and fathers in the period of post-outbreak rather than pre-outbreak is important, although this difference is not significant, considering the family-oriented culture of Iran, it is important to pay attention to the impact of couples’ participation in household chores and parenting responsibilities on MS (Rhyne, 1981; Korja et al., 2016; Liu and Wu, 2018; Leung, 2020; Yoo, 2020). In contextual explanation, as described in the descriptive analysis, engaging of parents in different activities, such as art and household activities, the extending of virtual relationships with family and relatives, the strengthening of religious beliefs, and asking help from supernatural powers, etc. can play an important role in reducing the impact of stressful events on family outcomes.

The results also showed that fathers had higher psychological well-being than mothers. Baruch et al. (1987) and World Health Organization [WHO] (2019) reported that women have poorer well-being due to more responsibilities of family based on unequal distribution of roles, as well as feeling less control over life. These studies have shown that mothers experience high stress and anxiety and low well-being due to the pressures of housekeeping and parenting responsibilities and high societal expectations of motherhood (Skreden et al., 2012; Musick et al., 2016; Meier et al., 2018; Nelson-Coffey et al., 2019). Consistent with this finding, another finding of this study showed that the mothers were more burned out than the fathers. Few evidences showed that burnout is higher in mothers or female caregivers than in fathers (Le Vigouroux and Scola, 2018; Lebert-Charron et al., 2018; Mikolajczak et al., 2018b). In many cultures, women play a permanent role in childcare. Excessive and uninterrupted childcare responsibilities, along with other responsibilities in life and trying to being a perfect mother, make them exhausted from parenting tasks, reduce their mental health, and cause emotional problems (Meeussen and Van Laar, 2018; Sánchez-Rodríguez et al., 2019; Sorkkila and Aunola, 2020).

The finding also revealed that fathers reported greater MS than mothers. This finding is consistent with many studies on gender differences in MS (Rhyne, 1981; Olson et al., 2008; Ogolsky and Bowers, 2013; Beam et al., 2018). Women are more concerned about intimacy than men, have certain standards for being satisfied with their spouses, and are sensitive to relationship problems more than men (Olson et al., 2008; Ayub and Iqbal, 2012).

The present study is one of the few surveys done in a limited time, so it is not possible to study the longitudinal effect of home quarantine (before, now, and after quarantine), Therefore, to overcome this limitation, parents’ perceptions of MS and PB in the pre- and post-prevalence period by asking questions in the form of past and present tenses were assessed; conducting the research nearly after 1 month and a half of prevalence and quarantine and its coincidence with the beginning of the New Year in Iran, type of home quarantine and not punished for leaving home, not focusing on living space and the children’s age due to focus on the number of children makes the researchers to be cautious in interpreting the findings. Therefore, to study parent–child backgrounds, increase sample size, pay attention to high-risk groups, and those who lost a member of the family to COVID-19 are recommended for further research.

## Data Availability Statement

The datasets presented in this study can be found in online repositories. The names of the repository/repositories and accession number(s) can be found below: https://osf.io/htu8p/.

## Ethics Statement

This research was conducted in accordance with the ethical codes of the Helsinki Declaration on the consent of the participants (in written) and their anonymity in the research.

## Author Contributions

The author confirms being the sole contributor of this work and has approved it for publication.

## Conflict of Interest

The author declares that the research was conducted in the absence of any commercial or financial relationships that could be construed as a potential conflict of interest.

## Footnotes

1 https://porsline.ir
